# A Novel Technique for Preparation of Recipient Site and Autologous Bone Grafting in Autotransplantation of Single-Rooted Teeth: A Report of Two Cases

**DOI:** 10.7759/cureus.31888

**Published:** 2022-11-25

**Authors:** Eduardo Anitua, Laura Piñas, Mohammad H Alkhraisat

**Affiliations:** 1 Regenerative Medicine Laboratory, Instituto Eduardo Anitua, Vitoria, ESP; 2 Clinical Research, Instituto Eduardo Anitua, Vitoria, ESP; 3 Regenerative Medicine, BTI Biotechnology Institute, Vitoria, ESP

**Keywords:** prgf, tooth vitality, periodontal healing, root development, low-speed drilling, tooth autotransplantation

## Abstract

Low-speed drilling without irrigation has a long history of use in implant dentistry. It provides the advantage of avoiding the wash-out of proteins and biomolecules from the bone. In this case report, we describe the novel use of this drilling protocol in the preparation of bone alveolus during the procedure of tooth autotransplantation. Two cases with early tooth loss in the upper maxillary arch were treated by the autotransplantation of permanent teeth with immature root development and the use of plasma rich in growth factors. Autologous bone fragments (from drilling) were harvested and used for alveolar bone augmentation. The follow-up time was three and seven years since tooth autotransplantation. All the autotransplanted teeth achieved a closed apex with no signs of loss of vitality. Low-speed drilling without irrigation did not jeopardize the outcomes of tooth autotransplantation and warrants further investigation in the context of periodontal ligament healing.

## Introduction

Dental implants as a prosthetic replacement of the dental root have demonstrated high success rates. Several longitudinal studies have reported survival rates of 90-95% over follow-up periods of 5-10 years [1]. In young patients, however, the ankylotic healing of dental implants may lead to infraocclusion. The risk of infraposition of dental implants (ankylosed) can be related to the growth of the alveolar bone height in an individual with ongoing vertical growth [2]. This results in the infraposition of the dental implant relative to the surrounding teeth. In the 1960s, Slagsvold and Bjercke described a protocol for the autotransplantation of teeth at the University of Oslo [3]. This article reported for the first time the procedure for performing tooth autotransplantation and the steps to be followed to perform the technique [3]. Since then, the technique has been widely accepted as an alternative to dental implants or other restorative treatments, especially in young patients. Tooth autotransplantation is especially indicated to replace missing teeth in children and adolescents but also is being applied in adults [4]. Tooth autotransplantation allows the formation of the periodontal ligament, the normal development of the alveolar bone, and the capacity for functional adaptation [3]. The success of tooth autotransplantation can be affected by different factors such as tooth type, root development, extraoral time, mechanical stress, and tooth stabilization, among others [5].

Since the technique has been described, it has undergone several modifications, mainly to achieve the best fit between the recipient bed and the morphology of the root of the autotransplanted tooth [6]. The tooth must be inserted in a new alveolus that adapts well to the root morphology, generating support for the tooth while revascularization and bone neoformation occur. The use of 3D-printed root replicas has been introduced to guide clinicians during the preparation of the bone alveolus [4]. Care should be paid not to excessively compress the developing apex. Therefore, the protocol for preparing the receptor bed when preparing the alveolus for the insertion of the transplanted tooth is crucial [6].

In implant dentistry, the dental implant success at the early stages of healing can be deteriorated by bone exposure to overheating and excessive trauma during the drilling of the alveolar bone [7]. Low-speed bone drilling without irrigation has been suggested to avoid the lavage of growth factors and biomolecules from the bone [8]. The isolation of human alveolar bone cells from the bone tissue harvested by this drilling protocol is indicative of its thermal safety that has not affected cellular viability [8]. These cells have shown characteristics of mesenchymal stem cells with osteogenic properties and a high proliferation rate [8].

The objective of this case series is to describe the implementation of low-speed drilling without irrigation in the procedure of tooth autotransplantation and the clinical outcomes.

## Case presentation

**Figure 1:**
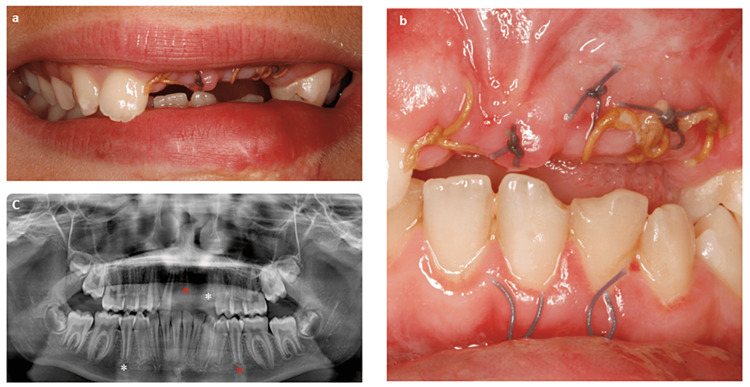
The clinical case at the patient’s first visit to the dental office. The patient had missing upper central incisors (#11 and #21), upper left lateral incisor (#22), and upper left canine (#23) (a-c). The lower second premolars (#35 and #45) had an open apex with incomplete root formation (c). *: Labeling the teeth to be autotransplanted and their future position.

The first patient was a 12-year-old female who, after a trauma caused by a bicycle accident, lost several teeth in the anterior-superior sector. The upper central incisors (#11 and #21), the upper left lateral incisor (#22), and the upper left canine (#23) were missing (Figure 1).

The lower second premolars (#35 and #45) had an open apex with incomplete root formation. The treatment plan was designed to restore the upper anterior sector by the autotransplantation of the lower second premolars at the position of the upper left central incisor (#21) and upper left lateral incisor (#22).

**Figure 2:**
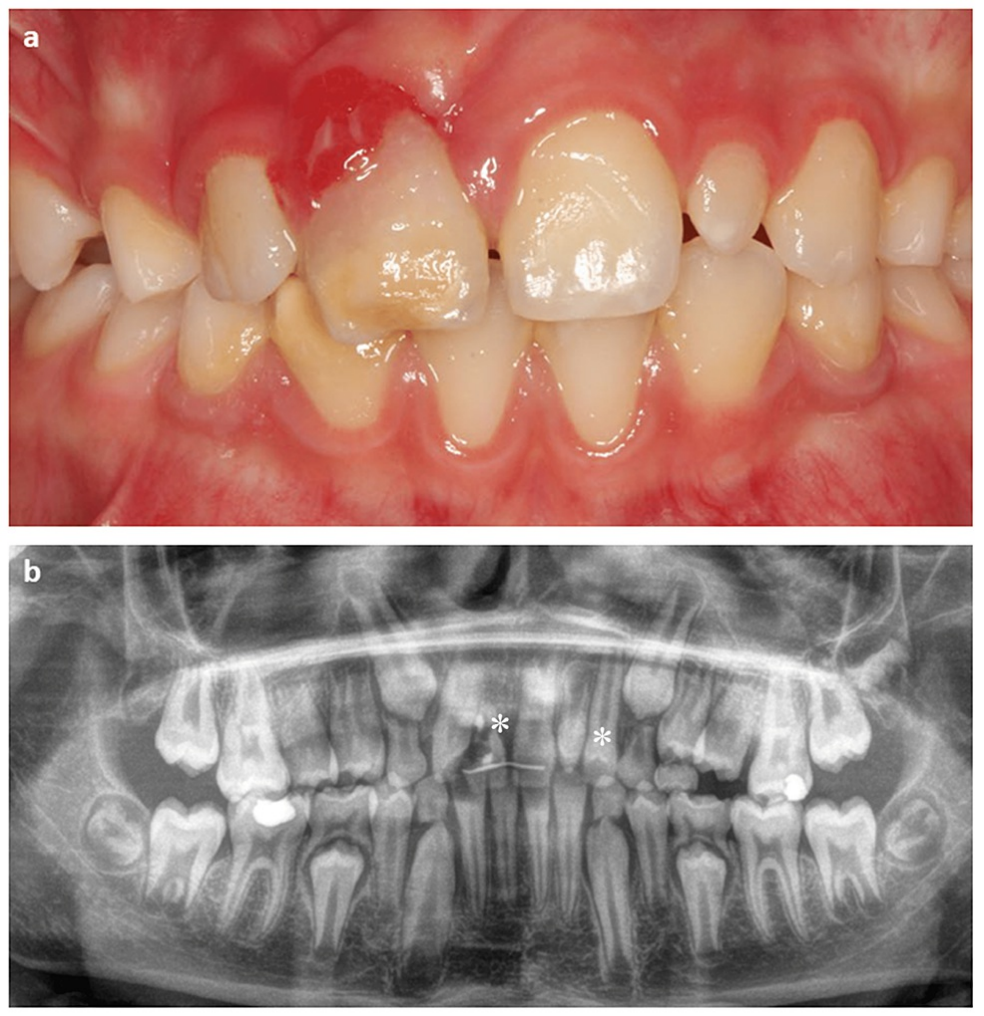
Internal resorption of the upper right central incisor (#11) (a and b). *: Indicates the tooth to be autotransplanted and its future position.

The second patient was an 11-year-old male who reported a history of trauma in the anterior sector. The clinical examination indicated the presence of root resorption in the upper right central incisor (#11). A supernumerary tooth was noted at the position of the upper left lateral incisor (#22) (Figure 2).

**Figure 3:**
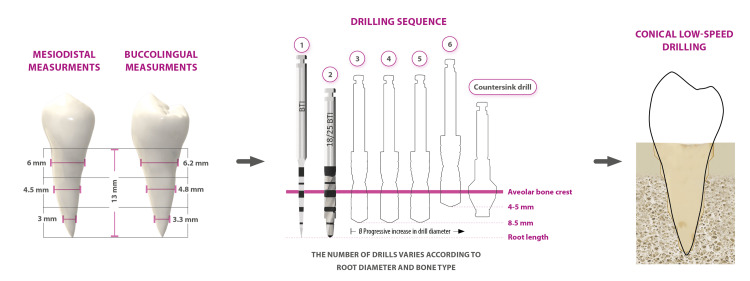
Scheme of the described technique for the preparation of an alveolus to receive the autotransplanted tooth. The initial drill was used at high speed with irrigation. The diameter drills were used at low speed (150 rpm) without irrigation. Image credits: Eduardo Anitua.

The treatment plan was designed to extract the upper right central incisor (#11) and replace it with the autotransplantation of the upper left lateral incisor (#22). The conoide supernumerary was conserved to replace the upper left lateral incisor (#22). The surgical preparation of the recipient sites was planned using measurements on the cone-beam computed tomography (CBCT) scan (3D visualization of the teeth and alveolar bone) of the teeth to be transplanted (Figure 3).

The diameter and length of each tooth were analyzed. The root was divided into coronal, medial, and apical thirds. The highest diameter of the root in each third was annotated and used to select the diameter of the drill for alveolus preparation. Under intravenous sedation, local anesthesia (4% articaine hydrochloride anhydrous with 1:200,000 adrenaline tartrate) was administered, and the alveolus was prepared following low-speed drilling without irrigation (150 rpm) except for the initial drill (850-1,000 rpm with copious irrigation) (Figure 3). Sharp new drills were used. Bone density at the recipient site was measured on the CBCT scan using software (BTI scan IV, BTI Biotechnology Institute, Vitoria, Spain). The length of the alveolus was divided into thirds and prepared using the drill with the same diameter as the root at each third (Figure 3).

Preoperatively, blood extraction into four anticoagulant-containing tubes (9 mL) (KMU 15; BTI Biotechnology Institute, Vitoria, Spain) was performed for the preparation of plasma rich in growth factors (PRGF). Briefly, after blood centrifugation (580 g for eight minutes) at room temperature, the plasma column was divided into fraction 1 (F1) and fraction 2 (F2) [9,10]. F2 was defined as 2 mL of plasma just above the buffy coat and F1 was the rest of the plasma column above the F2. For activation, 10% calcium chloride (PRGF activator) was used at a ratio of 20 µL per 1 mL of PRGF. The activation PRGF was kept at 37°C for clotting (8-10 minutes).

**Figure 4:**
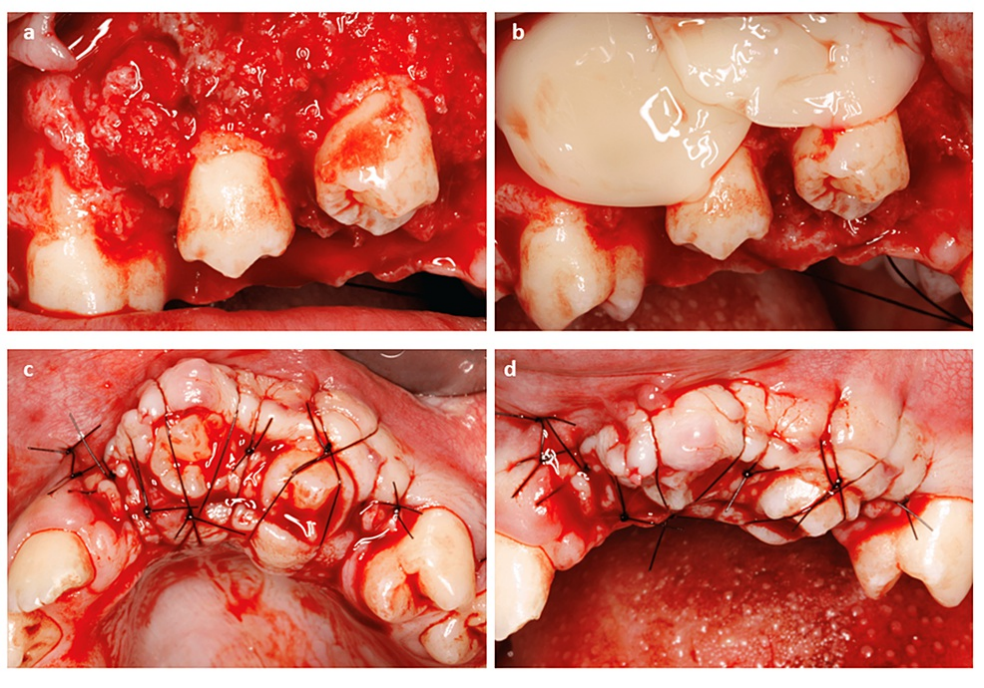
Clinical images of case one showing the placement of the autotransplanted teeth. Placement of the two autotransplanted lower second premolars and the bone augmentation of the alveolar bone on the vestibular wall (a), plasma rich in growth factors fibrin membranes covering the surgical area (b), anchorage of the teeth with suspensory stitches (c), and closure of the full-thickness flap (d). This stabilized the autotransplanted teeth during the healing period.

After the elevation of the full-thickness flap, the initial and pilot drills were used to mark the site of the alveolus at the total length of the root of the tooth to be autotransplanted. Diameter drills were then used to shape the alveolus. All bone harvesting by low-speed drilling was preserved in F2 of the PRGF without being activated with calcium chloride. After finishing the preparation of the alveolus, surgical extraction of the tooth for autotransplantation was carefully performed. The tooth was extracted causing minimal surgical trauma possible, whereby careful rotational dislocation movements are performed. The recipient alveolus was irrigated with F2 of the PRGF without activation and the autotransplanted tooth was inserted. The extra-alveolar time was always less than 30 seconds and successful insertion was achieved on the first attempt. Activated PRGF was then applied around the autotransplanted tooth. The tooth was stabilized by figure-of-eight crossing sutures that anchored it to the alveolar ridge. For bone augmentation on the labial side of the alveolar bone, the PRGF with autogenous bone was activated by 10% calcium chloride to induce clot formation. The sticky bone graft was then placed on the alveolar bone. The entire surgical area was covered with autologous fibrin membranes prepared from F1 of the PRGF after being activated, retracted (50% of the original size), and compressed (Figure 4). The membranes were stabilized by replacing and suturing the flap.

Subsequently, the flap was sutured, and suspensory sutures were placed. The 5-0 polyamide monofilament suture was used. Amoxicillin 500 mg three times a day and ibuprofen 400 mg three times a day were prescribed for one week. A 0.1% chlorhexidine mouth rinse was also prescribed for seven days. A soft diet was instructed for 48 hours and chewing with the autotransplanted teeth was prohibited. The sutures were removed after two weeks.

**Figure 5:**
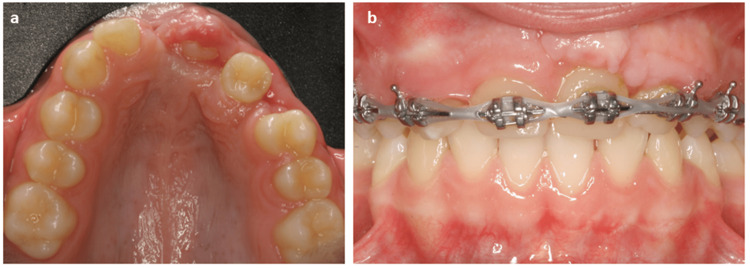
Case one follow-up. The clinical situation of case one at the end of the healing period (a) and at the orthodontic treatment (b). During the process, aesthetic contouring of the teeth was performed to achieve correct occlusion and aesthetics, transforming premolars into lateral and central incisors.

**Figure 6:**
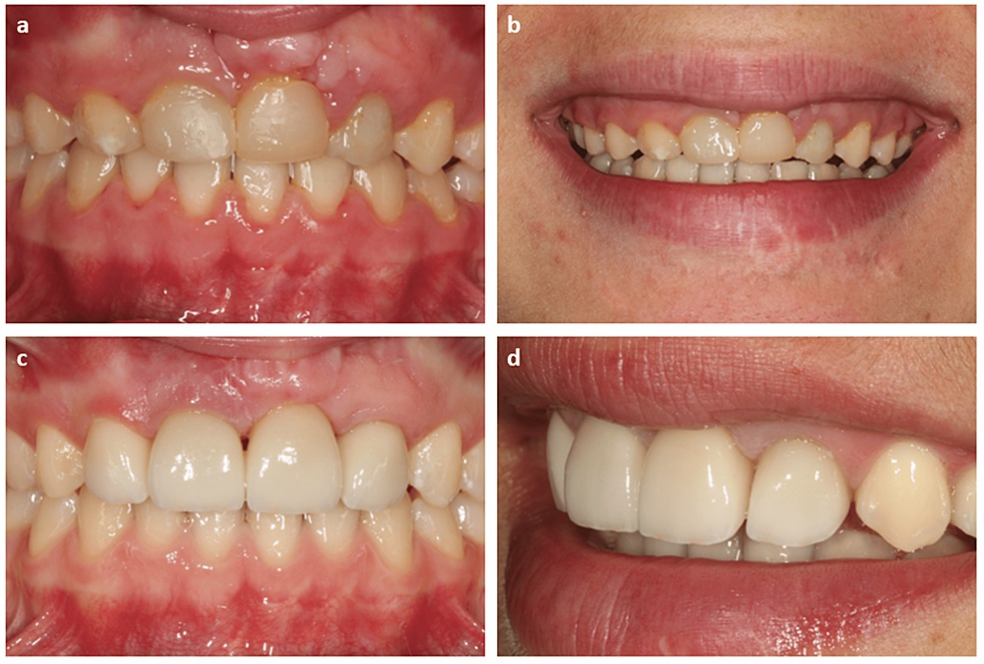
Long-term follow-up of case one. Clinical images of case one showing temporary aesthetic reconstructions after finishing the patient’s orthodontic treatment (a and b) and case evolution after seven years of follow-up, with aesthetic reconstructions carried out after the patient reached adulthood (c and d).

Clinical and radiographic examination was performed to assure that the tooth was asymptomatic and periodontally healthy. After six months of autotransplantation, orthodontic treatment was initiated to correct the occlusion and position the autotransplanted tooth in the dental arch (Figure 5). Fixed orthodontics with metallic braces were applied to correct the tooth position. The orthodontic treatment was finished in both cases in less than two years. Once they were moved to the corresponding area of the arch, aesthetic and functional rehabilitation of the autotransplanted tooth, wherever considered necessary, was performed (Figure 6). In both cases, porcelain veneers were used.

**Figure 7:**
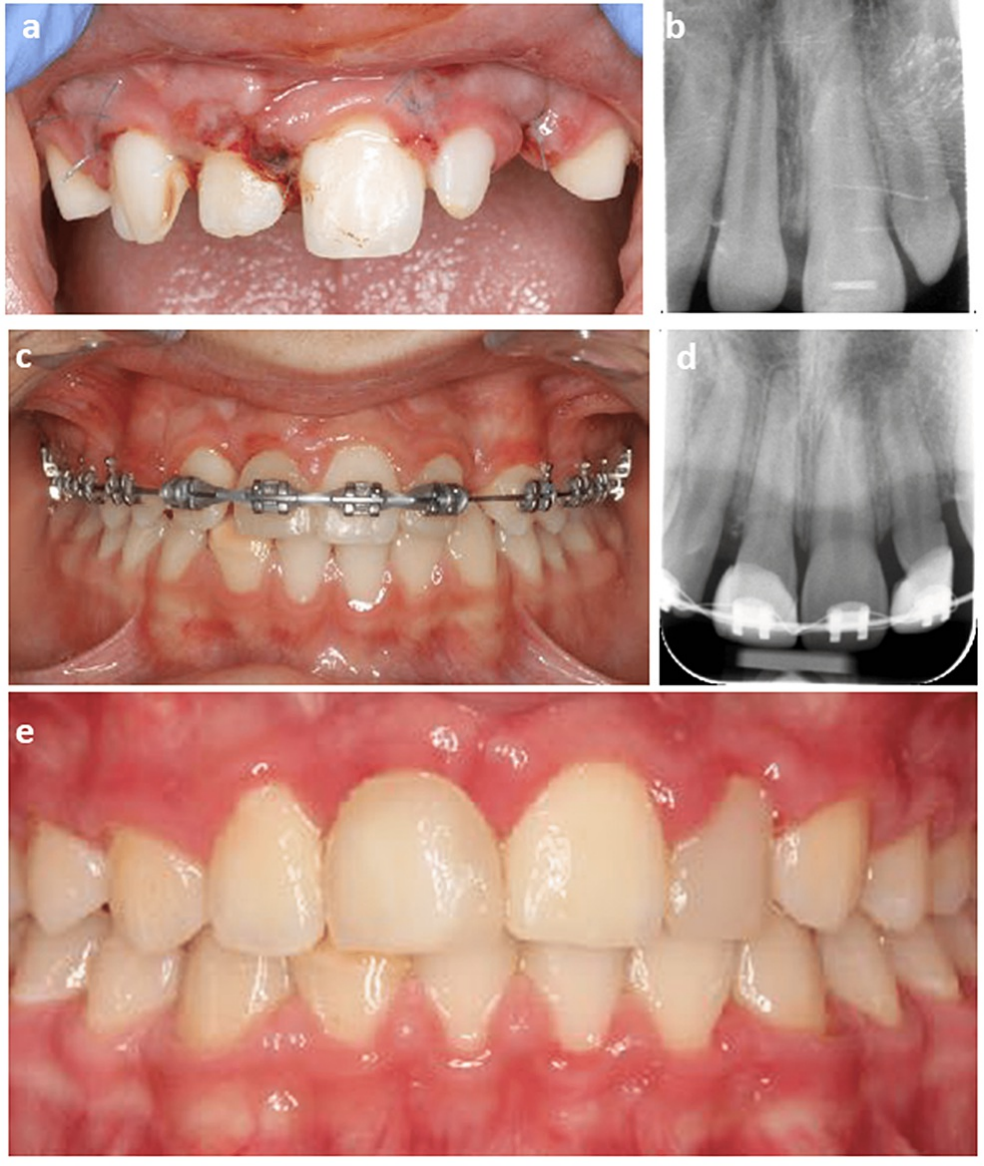
Clinical follow-up of case two. Clinical and radiographic images of case 2 showing the stabilization of the autotransplanted upper lateral incisor at the position of the upper right central incisor by splinting to the upper left central incisor (a), postoperative periapical radiograph at four days after tooth autotransplantation (b), the teeth position at the end of orthodontic treatment (c) and root formation with the closure of the apex of the autotransplanted tooth after one year (d), and the aesthetic reconstruction of the autotransplanted tooth (e).

Furthermore, root development was monitored to detect any signs of problems. Successful autotransplantation was indicated by the absence of inflammatory root resorption or apical periodontitis, and the roots of the autotransplanted tooth were completely formed (Figure 7).

## Discussion

Tooth autotransplantation is a versatile technique with a long history in dental practice for the rehabilitation of missing teeth in young patients where the insertion of implants cannot be carried out [3,5]. Other indications include the management of impacted or ectopic teeth [4]. In comparison to osseointegration, successfully autotransplanted teeth allow the growth and development of the alveolar bone, the eruption of the adjacent teeth, and the formation of new and vital periodontium [4,11]. The success of autotransplantation is sensitive to favorable healing of the periodontal ligament [11]. Care should be paid to avoid mechanical and/or biochemical damage to the periodontal ligament tissue [4]. For example, inadequate tooth extraction or alveolus preparation would place the periodontal ligament tissue under mechanical stress that may jeopardize healing and the success of tooth autotransplantation. Indeed, successful tooth autotransplantation is an outcome of adequate mechanical and biomechanical manipulation (extraoral environment) of the autotransplanted tooth. For that, it is plausible that the planification of the drilling protocol and the implementation of low-speed drilling without irrigation have generated adequate stimuli for successful outcomes. In implant dentistry, low-speed bone drilling without irrigation does not cause overheating of the bone [8]. The good quality of bone particles has confirmed the absence of bone damage [8]. Furthermore, periodontal ligaments have a major role in the healing of periodontal wounds. The periodontal ligaments have a high capacity for remodeling and renewal. The periodontal ligament fibroblasts are the predominant cell type and have osteoblast-like and fibroblast-like properties. This is important for periodontal regeneration which requires a coordinated formation of new cementum, new alveolar bone, and new periodontal ligament [12]. PRGF has significantly enhanced the proliferation, migration, and adhesion to collagen type I matrix of the periodontal ligament fibroblasts. It has also stimulated the synthesis of hepatocyte growth factor (anti-inflammatory and anti-fibrotic growth factor) and the connective tissue growth factor [13]. This factor is involved in several functions that include angiogenesis, cell proliferation, cell migration, cell adhesion, and extracellular matrix production.

Furthermore, the autotransplanted immature teeth in this study achieved root development and the formation of the closed apex. This is indicative of the presence of adequate blood supply and reservoir of stem cells that have resulted in the presence of vascularized connective tissue in the pulp that would induce root development and maintenance of tooth vitality [4]. Positive response to electric stimulus and the closure of root apex are expected outcomes for autotransplanted teeth with incomplete root development [4]. Furthermore, the use of PRGF has been effective in inducing the human dental pulp stem cell homing of root canal through a chemoattractant effect on the neighboring cells, as well as the stimulation of cell migration and proliferation [14]. The potential of PRGF to stimulate human dental pulp stem cell differentiation has been reported [15]. Furthermore, it provides a scaffold that supports cell homing of the root canal. It stimulates neoangiogenesis and the vascularization of the connective tissue [16]. In an inflammatory model using lipopolysaccharide, the use of platelet-rich plasma augmented the genetic expression of pro-angiogenic genes and adhesion molecule-related genes [17]. Moreover, it stimulated the expression of the cytokine CXCL1 (induces the migration of microvascular endothelial cells and tube formation) and suppressed the expression of IFNA1 (inhibits angiogenesis). PRGF is a pure platelet-rich plasma that contains no leukocytes that would induce inflammation and decreases the stability of the fibrin scaffold [14,16]. PRGF has anti-inflammatory properties that would enhance the balance toward tissue reconstruction and improve the quality of life of the patient [18]. PRGF has also shown antibacterial properties upon its activation to start clotting and is better resistant to bacteria-induced fibrinolysis [19]. Additionally, the filling of the socket with PRGF can (after clotting) help stabilize the tooth and impede the bacterial colonization of the gap (non-critical bone defect) between the socket walls and the autotransplanted tooth [18,20].

PRGF has been used with successful long-term outcomes in the autotransplantation of mature teeth [18]. It has been used as a scaffold for the revascularization of an autotransplanted mature tooth after the application of the fragile fracture technique.

The study has several limitations. The study describes cases where the autotransplanted tooth has an open apex and no information is yet available on its use when the tooth is mature with a closed apex. Providing an equilibrium between the primary stability and the compression of the periodontal ligament is needed. The lack of a control group regarding the drilling speed and the use of PRGF is another limitation of this study. More studies with larger sample sizes are needed.

## Conclusions

Within the limitation of this case report, the use of low-speed drilling without irrigation to prepare the alveolus for tooth autotransplantation has resulted in successful outcomes. The bone fragments can be collected and used as graft material for the overcorrection of the alveolar ridge. The contribution of autologous PRGF in the enhancement of the probability of successful autotransplantation needs further investigation.

## References

[1] Esposito M, Hirsch JM, Lekholm U, Thomsen P (1998). Biological factors contributing to failures of osseointegrated oral implants. (II). Etiopathogenesis. Eur J Oral Sci.

[2] Klinge A, Tranaeus S, Becktor J, Winitsky N, Naimi-Akbar A (2021). The risk for infraposition of dental implants and ankylosed teeth in the anterior maxilla related to craniofacial growth, a systematic review. Acta Odontol Scand.

[3] Slagsvold O, Bjercke B (1978). Applicability of autotransplantation in cases of missing upper anterior teeth. Am J Orthod.

[4] Plotino G, Abella Sans F, Duggal MS, Grande NM, Krastl G, Nagendrababu V, Gambarini G (2022). Present status and future directions: surgical extrusion, intentional replantation and tooth autotransplantation. Int Endod J.

[5] Paulsen HU, Andreasen JO, Schwartz O (1995). Pulp and periodontal healing, root development and root resorption subsequent to transplantation and orthodontic rotation: a long-term study of autotransplanted premolars. Am J Orthod Dentofacial Orthop.

[6] Czochrowska EM, Stenvik A, Bjercke B, Zachrisson BU (2002). Outcome of tooth transplantation: survival and success rates 17-41 years posttreatment. Am J Orthod Dentofacial Orthop.

[7] Gapski R, Wang HL, Mascarenhas P, Lang NP (2003). Critical review of immediate implant loading. Clin Oral Implants Res.

[8] Anitua E, Troya M, Zalduendo M, Flores J, Tierno R, Alkhraisat MH (2020). The influence of alveolar bone healing degree on its potential as a source of human alveolar bone-derived cells. Ann Anat.

[9] Anitua E, Murias-Freijo A, Alkhraisat MH, Orive G (2015). Clinical, radiographical, and histological outcomes of plasma rich in growth factors in extraction socket: a randomized controlled clinical trial. Clin Oral Investig.

[10] Anitua E (1999). Plasma rich in growth factors: preliminary results of use in the preparation of future sites for implants. Int J Oral Maxillofac Implants.

[11] Lacerda-Santos R, Canutto RF, Araújo JL (2020). Effect of orthodontic treatment on tooth autotransplantation: systematic review of controlled clinical trials. Eur J Dent.

[12] Volponi AA, Pang Y, Sharpe PT (2010). Stem cell-based biological tooth repair and regeneration. Trends Cell Biol.

[13] Bendinelli P, Matteucci E, Dogliotti G, Corsi MM, Banfi G, Maroni P, Desiderio MA (2010). Molecular basis of anti-inflammatory action of platelet-rich plasma on human chondrocytes: mechanisms of NF-κB inhibition via HGF. J Cell Physiol.

[14] Widbiller M, Driesen RB, Eidt A (2018). Cell homing for pulp tissue engineering with endogenous dentin matrix proteins. J Endod.

[15] Anitua E, Zalduendo M, Troya M (2019). Autologous plasma rich in growth factors technology for isolation and ex vivo expansion of human dental pulp stem cells for clinical translation. Regen Med.

[16] Anitua E, Zalduendo M, Troya M, Tierno R, Alkhraisat MH (2022). The inclusion of leukocytes into platelet rich plasma reduces scaffold stability and hinders extracellular matrix remodelling. Ann Anat.

[17] Bindal P, Gnanasegaran N, Bindal U, Haque N, Ramasamy TS, Chai WL, Kasim NH (2019). Angiogenic effect of platelet-rich concentrates on dental pulp stem cells in inflamed microenvironment. Clin Oral Investig.

[18] Gaviño Orduña JF, García García M, Dominguez P, Caviedes Bucheli J, Martin Biedma B, Abella Sans F, Manzanares Céspedes MC (2020). Successful pulp revascularization of an autotransplantated mature premolar with fragile fracture apicoectomy and plasma rich in growth factors: a 3-year follow-up. Int Endod J.

[19] Drago L, Bortolin M, Vassena C, Taschieri S, Del Fabbro M (2013). Antimicrobial activity of pure platelet-rich plasma against microorganisms isolated from oral cavity. BMC Microbiol.

[20] Torres J, Tamimi FM, Tresguerres IF, Alkhraisat MH, Khraisat A, Lopez-Cabarcos E, Blanco L (2008). Effect of solely applied platelet-rich plasma on osseous regeneration compared to Bio-Oss: a morphometric and densitometric study on rabbit calvaria. Clin Implant Dent Relat Res.

